# Host-Associated Rhizobial Fitness: Dependence on Nitrogen, Density, Community Complexity, and Legume Genotype

**DOI:** 10.1128/aem.00526-22

**Published:** 2022-07-19

**Authors:** Liana T. Burghardt, Brendan Epstein, Michelle Hoge, Diana I. Trujillo, Peter Tiffin

**Affiliations:** a Department of Plant and Microbial Biology, University of Minnesota, St. Paul, Minnesota, USA; b Plant Science Department, The Pennsylvania State University, University Park, Pennsylvania, USA; c Department of Plant Pathology, University of Minnesota, St. Paul, Minnesota, USA; Georgia Institute of Technology

**Keywords:** nitrogen addition, inoculation density, community complexity, *Sinorhizobium (Ensifer) meliloti*, legume-rhizobia, strain relative fitness, *Medicago truncatula*, genotype × environment interaction, host-microbe interaction, nodules

## Abstract

The environmental context of the nitrogen-fixing mutualism between leguminous plants and rhizobial bacteria varies over space and time. Variation in resource availability, population density, and composition likely affect the ecology and evolution of rhizobia and their symbiotic interactions with hosts. We examined how host genotype, nitrogen addition, rhizobial density, and community complexity affected selection on 68 rhizobial strains in the Sinorhizobium meliloti*–*Medicago truncatula mutualism. As expected, host genotype had a substantial effect on the size, number, and strain composition of root nodules (the symbiotic organ). The understudied environmental variable of rhizobial density had a stronger effect on nodule strain frequency than the addition of low nitrogen levels. Higher inoculum density resulted in a nodule community that was less diverse and more beneficial but only in the context of the more selective host genotype. Higher density resulted in more diverse and less beneficial nodule communities with the less selective host. Density effects on strain composition deserve additional scrutiny as they can create feedback between ecological and evolutionary processes. Finally, we found that relative strain rankings were stable across increasing community complexity (2, 3, 8, or 68 strains). This unexpected result suggests that higher-order interactions between strains are rare in the context of nodule formation and development. Our work highlights the importance of examining mechanisms of density-dependent strain fitness and developing theoretical predictions that incorporate density dependence. Furthermore, our results have translational relevance for overcoming establishment barriers in bioinoculants and motivating breeding programs that maintain beneficial plant-microbe interactions across diverse agroecological contexts.

**IMPORTANCE** Legume crops establish beneficial associations with rhizobial bacteria that perform biological nitrogen fixation, providing nitrogen to plants without the economic and greenhouse gas emission costs of chemical nitrogen inputs. Here, we examine the influence of three environmental factors that vary in agricultural fields on strain relative fitness in nodules. In addition to manipulating nitrogen, we also use two biotic variables that have rarely been examined: the rhizobial community's density and complexity. Taken together, our results suggest that (i) breeding legume varieties that select beneficial strains despite environmental variation is possible, (ii) changes in rhizobial population densities that occur routinely in agricultural fields could drive evolutionary changes in rhizobial populations, and (iii) the lack of higher-order interactions between strains will allow the high-throughput assessments of rhizobia winners and losers during plant interactions.

## INTRODUCTION

Biotic interactions have important consequences for population dynamics (1), selection (2), and local adaptation (3) of interacting species. Of course, these biotic interactions do not occur in isolation and the benefits and costs of the interaction can be modified (4) by population density, the presence of additional species (5), genetics (6), and abiotic factors such as resource availability (7–10) or moisture levels (11–13). These responses can evolve if there is genetic variation in traits that modify the sensitivity of an interaction to additional environmental variables such as immunity, stress tolerance, or phenology (e.g., Ramegowda and Senthil-Kumar [14] and Garrido-Oter et al. [15]). Here we examine how three sources of environmental variation, resource availability, rhizobial density, and rhizobial community complexity, affect the rhizobium species Sinorhizobium meliloti as it engages in symbiosis with its leguminous host plant (Medicago truncatula).

Environment dependence is a recurring theme in the study of the symbiosis between rhizobial bacteria and legume plants (16–18). In this mutualistic relationship, rhizobia convert atmospheric nitrogen (N_2_) into a plant-useable form to support host growth and reproduction while rhizobia gain carbon resources from the plant to support the growth and reproduction prior to release back into the soil (19, 20). While this relationship is commonly beneficial to the plant host (21), the magnitude of these benefits to the plant depends on the identity of the rhizobial strain as well as additional environmental parameters including nitrogen (N), phosphorous, water and light availability, and temperature (17, 22–24). Experiments in which plants are inoculated with a single rhizobium strain, often at a very high density, have shown that the benefits rhizobia obtain from symbiosis also can be context dependent (e.g., Batstone et al. 2020 [25] and Friel and Friesen [26]). However, in natural and agricultural populations rhizobial densities are likely to vary and multiple rhizobial strains compete for nodule occupancy and host enrichment (18). Results from single-strain experiments may not be directly translatable to multistrain environments because between-strain competition can strongly affect nodulation success (27). Indeed, strain fitness proxies in single-strain environments are not strongly correlated with strain fitness in multistrain communities (28, 29).

Theoretically, resource availability has the potential to shape the evolution of resource-based symbiosis (30, 31). N availability could affect selection acting on rhizobia and plant hosts via a number of mechanisms. For instance, additional N can reduce the overall frequency of associations with hosts by reducing nodule number or size (16, 32, 33). While forming fewer nodules in high N environments may have little effect on plant fitness, it certainly reduces the chances of each rhizobium associating with a legume and, if nodules remain small, reduces the number of rhizobia released from host nodules (a major component of the fitness benefit rhizobia receive from engaging in the symbiosis). Nitrogen can also alter competitiveness among symbionts, perhaps through altering the strength of host preference (34) or enrichment via host rewards/sanctions (35, 36). Despite the appeal of theoretical predictions that additional N will reduce host dependence on rhizobia and lead to relaxed selection for rhizobial host benefit, there is limited empirical support for N-mediated shifts in competitive outcomes between rhizobia. Partner choice as measured by nodule occupancy is not strongly affected by N addition in *Lotus* (37), *Acmispon* (38), or *Medicago* (39, 40) and only weakly shifted in *Mimosa* (34). However, which strain initiates each nodule only represents the first stage of selection. Once nodules form, differential nodule growth and strain reproduction can allow some strains to increase in frequency relative to others, but again, studies suggest that N addition has only a limited effect on rhizobial relative fitness (24, 37, 39).

Unlike N addition, the effect of rhizobial population density on legume benefit and rhizobial fitness has received scant empirical attention. However, population densities can strongly affect the ecology and evolution of biotic interactions including hosts and pathogens (e.g., Schuhegger et al. [41]), predator and prey (e.g., Jaffee [42]), and plants and pollinators (e.g., Moeller [43]). The density of rhizobial symbionts varies over space and time. For example, in an agroecosystem, 20 years of cropping system differences resulted in four-orders-of-magnitude differences in rhizobia population densities: 6.8 × 10^6^ rhizobia gram^−1^ in soy/wheat/maize rotations, 4.5 × 10^5^ in continuous soy, and 6.1 × 10^2^ in continuous maize (44). Many possible mechanisms could lead to density-dependent rhizobial selection. For instance, the density of rhizobia in the soil could affect the number of nodules that a host forms (45); trigger a quorum sensing mechanism with cascading effects on exopolysaccharide production, plasmid transfer, and motility (46–48); or alter the relative importance of host-mediated versus soil-mediated selection (49, 50). However, predicting the directional outcome of rhizobial density on strain selection is difficult. For instance, when rhizobial population densities are high, we might expect plant-imposed selection on bacterial populations to increase as more nodules are formed overall and more nodulation sites (root hairs) directly interact with multiple strains (50). On the other hand, when rhizobial densities are high, plant control may decrease as traits that influence rhizobial competitiveness (e.g., motility and chemotaxis) become more important (27).

Much of the empirical work on legume-rhizobia symbiosis relies on single-strain inoculations; however, in nature legume hosts often form nodules with a diverse community of strains (51, 52). Microbial community complexity can affect community assembly and interactions (53). Synthetic communities are increasingly being used to query the effect of additional community members (54–56). For example, Friedman et al. (57) showed that competitive outcomes between two bacterial strains living in the guts of Caenorhabditis elegans are not affected by the presence of additional strains (58). While rhizobial strain frequency in nodules is clearly dependent on the presence of other strains (27, 39, 50), experiments have not investigated whether pairwise competitive outcomes are affected by the presence of other strains. In other words, does strain A always beat strain B regardless of which and how many other strains are present in the community?

**FIG 1:**
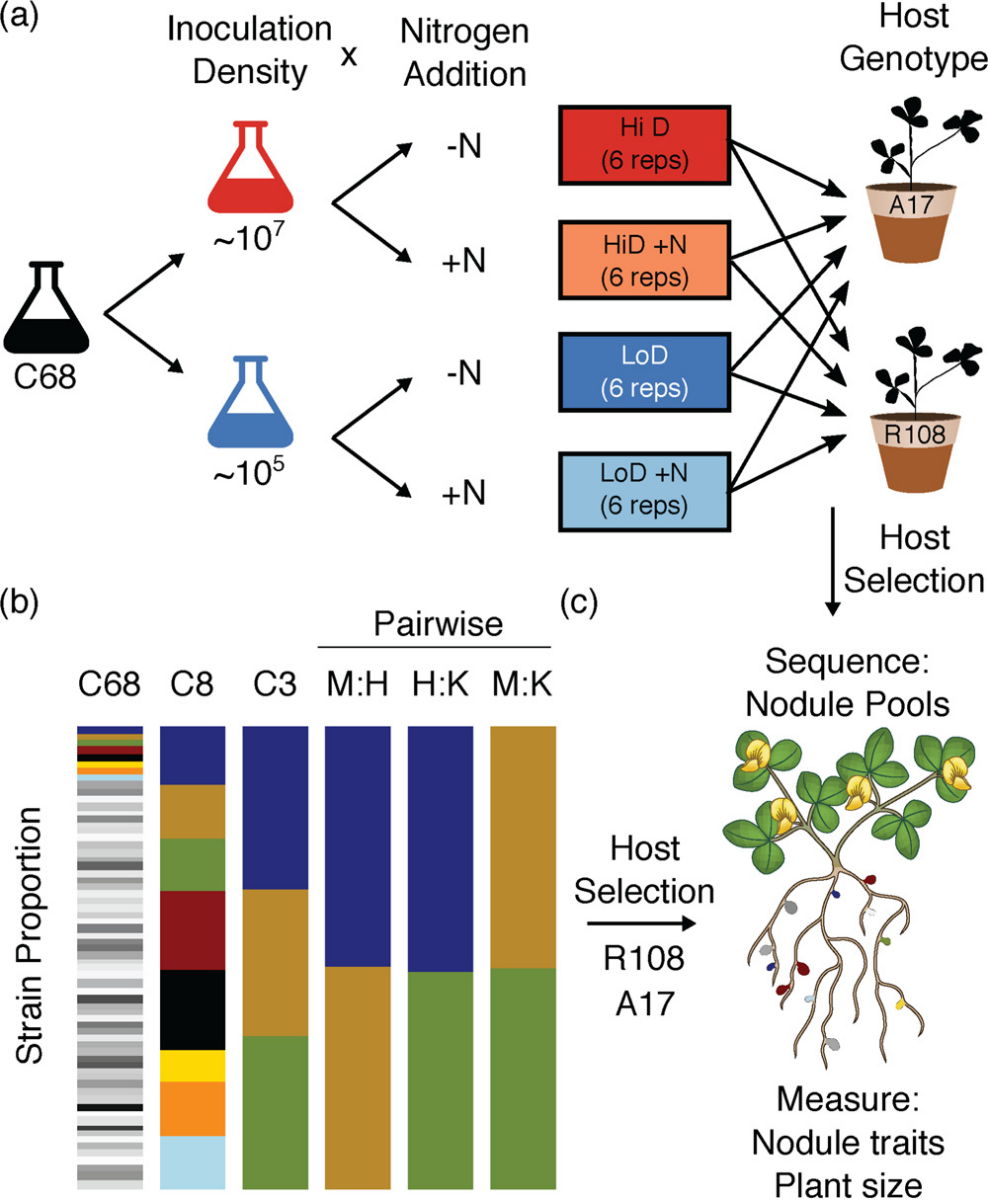
Design of the Nitrogen × Density (a) and Community complexity (b) experiments and traits measured on strains or plants in both experiments (c). All plants were harvested ~6 weeks after planting. Inoculation density is given per plant (~10 plants per pot). Colored stacked bars represent strain frequencies in each of six initial communities: 68 strains (C68: six replicate pots for each N × D treatment), eight strains (C8: five reps), three strains (C3: five reps), and two strain pairwise competitions (M:H, H:K, and M:K: four reps each).

Here we report on the extent to which rhizobial strain fitness in nodules and plant traits are affected by each of three environmental factors, N availability, population density, and the complexity of the rhizobial community to which plants are exposed (Fig. 1). We measured strain communities inside host nodules using a select-and-resequence approach, a variant of evolve-and-resequence approaches (28). We inoculated plants with a community of 68 strains of the rhizobium Sinorhizobium meliloti (hereafter referred to as C68). These strains were chosen to capture the majority of genetic variation present among 160 sequenced strains of S. meliloti from a worldwide collection of strains sampled across a broad range of environments (e.g., *Medicago* host species, geographic locals, and years) (59, 60). To evaluate whether increasing the amount of N available to plants altered the strain composition of the nodule community, we grew plants in additional N. At very high N levels many legumes including *Medicago* strongly reduce investment in nodules (numbers and nodule size), which makes an assessment of relative strain fitness in nodules difficult. We chose a level of nitrogen addition that reduces the plant’s dependence on rhizobia for nitrogen, yet ensures the formation of enough nodules to quantify strain fitness and ensure the statistical power to overcome the stochastic aspects of nodule formation. To each N-addition pot, we added 100 mL of a 3-mM KNO_3_ solution per week. All other pots received an equivalent amount of sterile water. To evaluate the potential for rhizobial density to affect strain fitness, we inoculated plants with two rhizobial densities: low (5 × 10^5^ rhizobia per plant) and high (5 × 10^7^ rhizobia per plant) (Fig. S1). We chose these levels to mimic rhizobial densities found in soils with established natural or agricultural legume populations. To examine the effect of community complexity, we constructed communities of nested subsets of eight, three, and all pairwise competitions of the three. Given work showing that rhizobial fitness can strongly depend on the host genotype (28, 61–63), we examined the effect of these treatments on each of two commonly used plant genotypes. Because N addition has been widely studied, it provides a nice contrast to less studied environmental factors.

Taken together, our results indicate that host-genotype has a much greater effect on strain fitness than environmental manipulations. Among the environmental manipulations, the effects of bacterial density were approximately twice as great as the effects of N addition. Community complexity had little effect on the relative fitness rankings of rhizobial strains suggesting that higher-order interactions between strains are rare in the context of host nodule formation. Our results suggest that variation in population densities could influence ecological and evolutionary dynamics in rhizobial communities and are a factor that should be considered more explicitly in empirical, theoretical, and applied work.

## RESULTS

### Host genotype has a large effect on strain fitness and nodule phenotypes.

**FIG 2:**
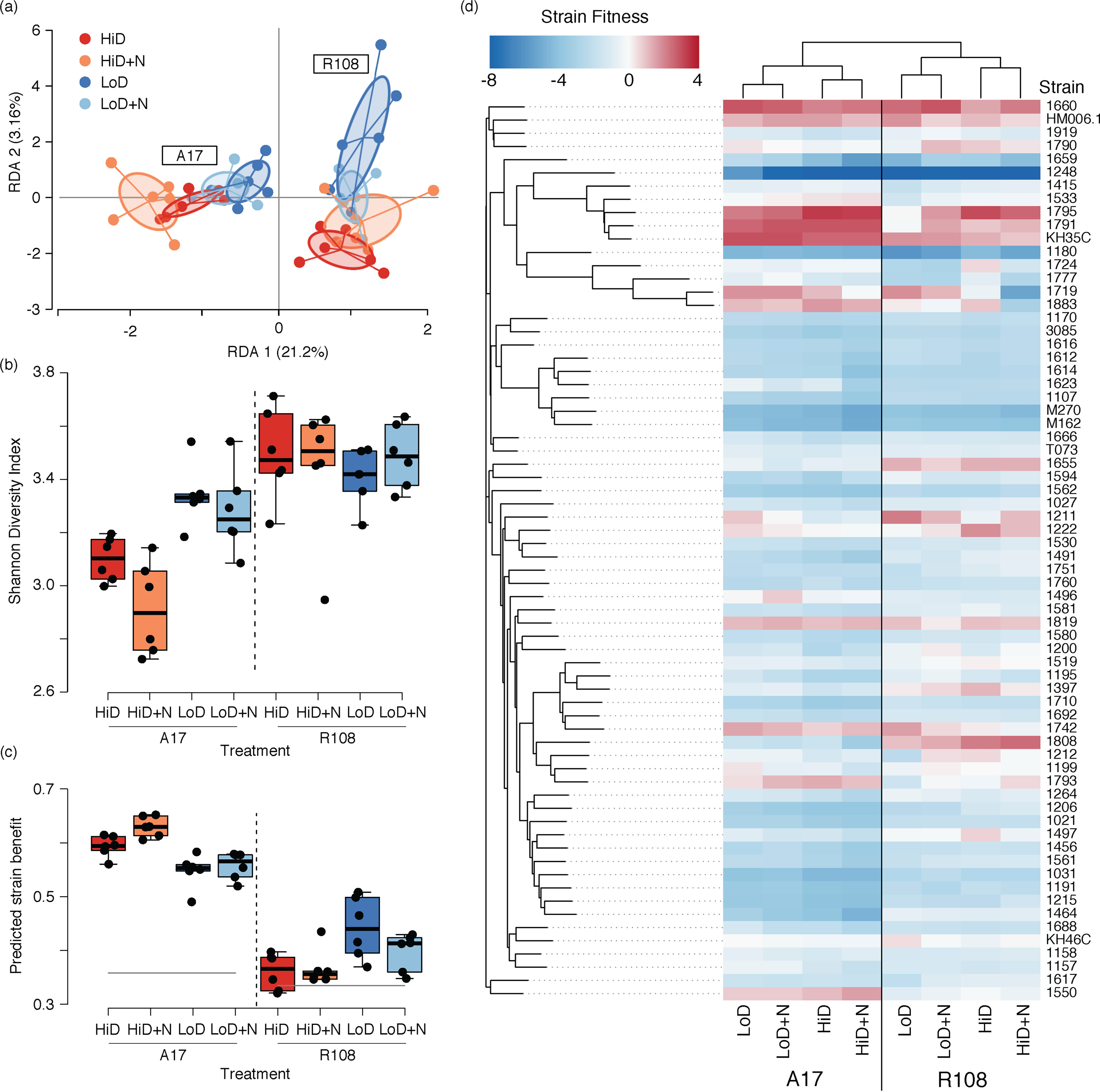
Host genotype and inoculum density affected rhizobia strain community characteristics. (a) Host genotype, density, and their interaction all contributed to variation in strain composition in nodules (percent contribution is in parenthesis [Table 1; Fig. S4] for RDA analysis by host genotype). (b) Shannon’s diversity decreased (i.e., stronger selection) at high densities (*P < *0.001) and high nitrogen (*P = *0.039) in A17. R108 nodules were always more diverse than A17 (*P < *0.001) regardless of density or nitrogen (Table S2 for full model). (c) N addition had little effect on the predicted benefit of nodule communities. However, predicted benefit significantly increased at high densities in A17 (*P < *0.001) and significantly decreased in R108 (*P = *0.003, full results in Table S3). (d) Heatmap of median strain fitness values. Strains (rows) are arranged by shared SNPs and treatments (columns) via hierarchical clustering.

**FIG 3:**
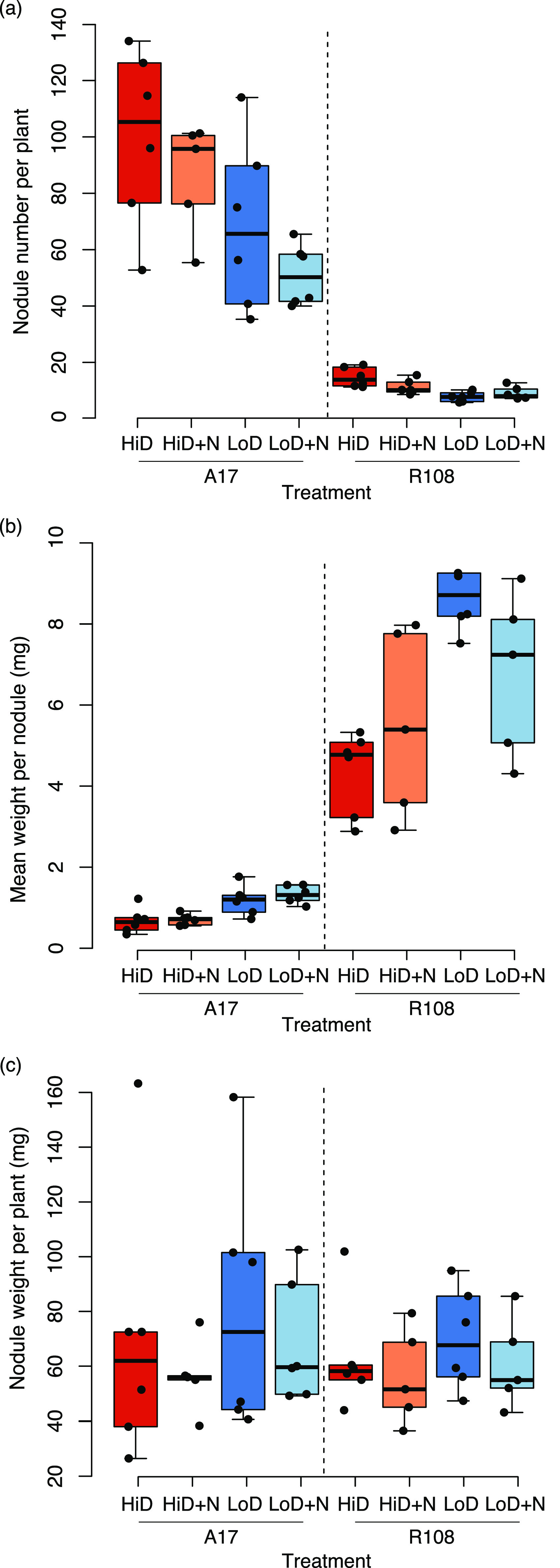
Nodule traits in Density (D) and Nitrogen (N) treatments. Hosts produced different numbers (a) and sizes (b) of nodules but a similar overall weight (c) across treatments. At low inoculation densities, nodule number decreased, and nodule weight increased (full results in Table S2).

Consistent with previous work on the same strains (64), with an overlapping set of strains (28, 63), and with an independent collection of rhizobial strains (29), the strain composition of the nodule communities was strongly affected by host genotype (Fig. 2 and Fig. S2). Host identity had a greater effect on strain composition, Shannon’s diversity, and predicted benefit of the nodule community (Fig. 2, Table 1, Table S1, and Table S2), than did N addition, inoculum density, or the complexity of the inoculum community. Relative to R108, A17 hosts produced ~10 times more nodules (Fig. 3a) that were approximately one-tenth the size (Fig. 3b) and harbored nodule strain communities that were less diverse (Fig. 2b) and more beneficial (Fig. 2c).

**TABLE 1:** PERMANOVA on RDA of strain relative fitness shows effects of plant host and inoculum density are more robust than effects of nitrogen (Both Hosts)^*a*^

Model and terms	DF	Prop. Var	*F*	*P*
Both Hosts (adj. *r*^2^ = 0.35)				
Host	1	0.275	19.81	**<0.001**
Density	1	0.056	4.02	**0.003**
Nitrogen	1	0.019	1.35	0.172
Host × Density	1	0.038	2.74	**0.015**
Host × Nitrogen	1	0.023	1.67	0.105
Density × Nitrogen	1	0.018	1.27	0.223
Host × Density × Nitrogen	1	0.016	1.13	0.272
Residual	40	0.556		
A17 only (adj. *r*^2^ = 0.20)				
Density	1	0.217	6.24	**<0.001**
Nitrogen	1	0.062	1.78	0.079
Density × Nitrogen	1	0.027	0.76	0.656
Residual	20	0.695		
R108 only (adj. *r*^2^ = 0.05)				
Density	1	0.069	1.68	**0.034**
Nitrogen	1	0.045	1.08	0.336
Density × Nitrogen	1	0.059	1.43	0.083
Residual	20	0.827		

a *P*-values less than 0.05 are formatted in bold. Analyses of each host separately (A17 only and R108 only) revealed that density and nitrogen had more significant effects with A17 than R108 hosts. Nitrogen was retained as a term in the submodels because host values and variances were so divergent from each other. DF, degrees of freedom; Prop. Var, proportion variance explained.

### N availability weakly affected strain composition, diversity, and benefit.

Because nitrogen is the primary resource rhizobia provide to their host, researchers have speculated and modeling has shown that N availability could alter the relative fitness of rhizobial strains. However, we found that N availability explained only a small portion of the variance in the overall composition of the nodule communities (1.9% of the redundancy analysis [RDA] variance, *P = *0.17; Fig. 2), although the effect was slightly greater when hosts were analyzed separately (in A17 *P = *0.079, 6.2% of the variance; and in R108 *P = *0.336, 4.5% of the variance; Table 1 and Fig. S3). N addition had a similar magnitude of effect on the diversity (Shannon’s) of the nodule community and the predicted benefit of the strain community, with the effects being greater in A17 (diversity: *P = *0.039, 8.7% of variance; and strain benefit: *P = *0.030, 7.9% of variance) than R108 (*P = *0.85, 0.17% of variance and *P = *0.28, 3.2% of variance, respectively). See Table S1 and Table S2 for full results.

### Rhizobial inoculation density had a more substantial effect than nitrogen addition.

Inoculating plants with 100-fold fewer rhizobial cells caused larger changes in community composition, strain diversity, and predicted benefit of the nodule community than N addition (Fig. 2). Moreover, the effect of density depended on the host. With A17 hosts, density explained 21.7% (*P = *0.001) of variance in community composition versus 6.2% (*P = *0.079) of the variance explained by N addition (Fig. S3b). With R108, density explained 6.9% (*P = *0.034) of the variance versus 4.5% (*P = *0.336) explained by N addition (Fig. S3c). Strain fitness shifts in response to density are so strong they are clear even in an unconstrained principal-component analysis (PCA) analysis of strain fitness (Fig. S4). Density affected the diversity of the nodule community in opposite directions in the two hosts. Compared to the low-density inoculation, the high-density inoculation resulted in A17 nodule communities being less diverse (based on Shannon’s diversity) and more beneficial (both *P < *0.001). By contrast, with R108, the low-density inoculation resulted in a less beneficial nodule community (*P < *0.003; Table S1 and S2).

### Relative fitness rankings are similar across communities of increasing complexity.

**FIG 4:**
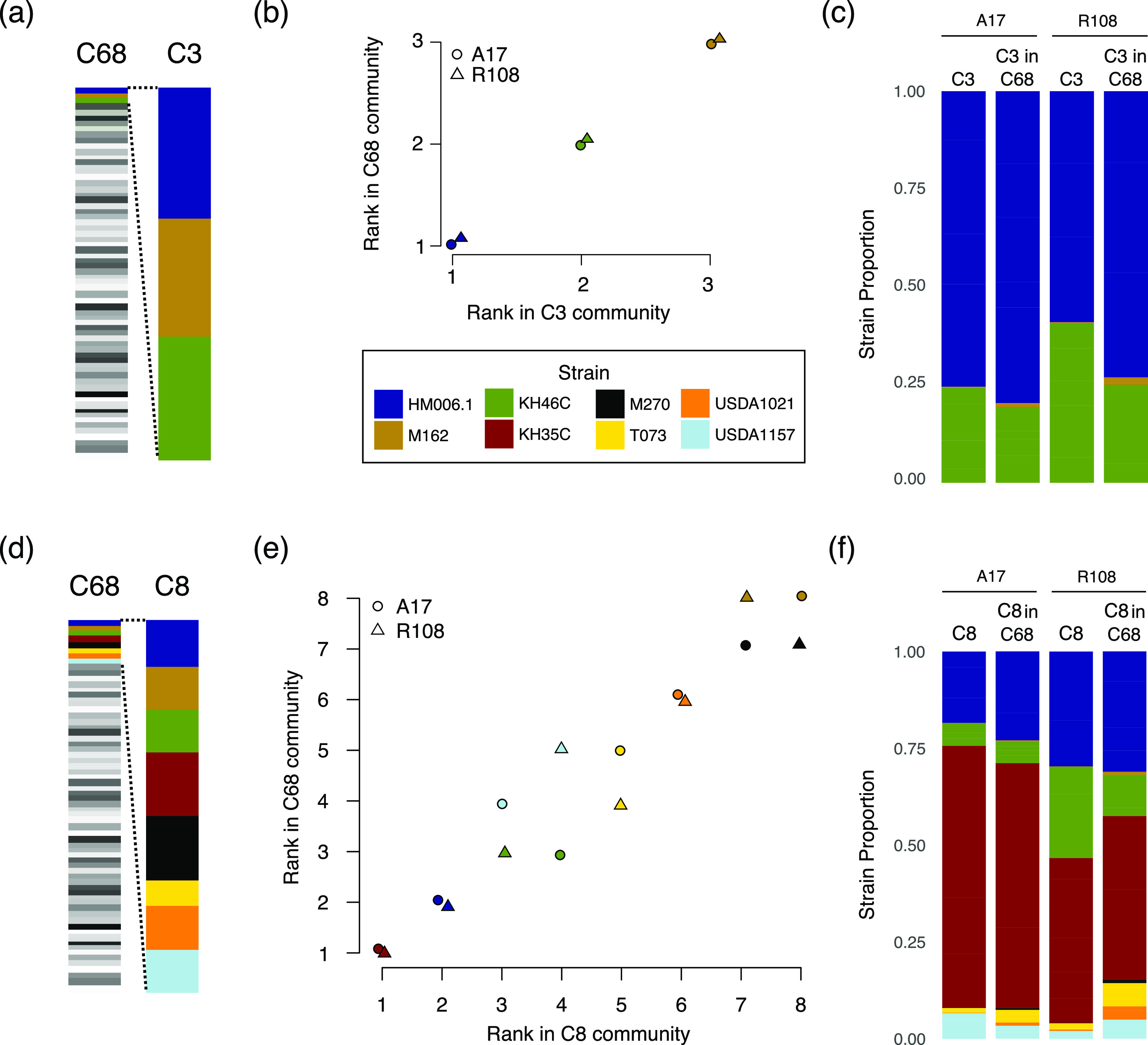
(a to f) Rank order of relative strain frequencies in the nodule communities were identical when plants were inoculated with either the 3-strain (C3) or 68-strain (C68) community (a to c) and highly similar when inoculated with either the 8-strain (C8) or C68 community (d to f). (a and d) show nested strain subsets of the initial communities. (b and e) Strain ranks as determined based on mean strain frequency across five (C3 and C8) or six (C68) replicates for A17 (triangles) and R108 (circles) and depicted in panels c and f. In A17, the third and fourth-ranked strains USDA1157 and KH46C swapped places, and in R108, T073, and USDA1157 swapped and the rarest strains M270 and M162 swapped. See Fig. S5 for additional contrasts and Fig. S6 for variation between individual replicates.

In nature, the complexity of rhizobial communities varies in both space and time. We found that although the absolute frequency of each of the three focal strains was strongly affected by the presence of additional strains in the inoculum, we found little evidence that additional strains in the inoculum community altered strain rankings relative to each other. In other words, with minor exceptions, the more frequent strain in pairwise competitions was also the more frequent strain in more complex communities (Table 2). The minor exceptions involved two strains (KH46c and HM006.1) that had nearly equal frequencies in pairwise inoculations and nearly equal frequency when part of more strain-rich communities (Fig. S5 and S6; Table 2). Similarly, the relative-frequency ranking of strains when plants were inoculated with three strains (C3) was almost always the same as the relative ranking of those strains when they were part of a 68-strain inoculum community (Figure 4a to c) and the relative ranking of the strains in C8 treatment was nearly always the same as in the C68 treatment (Figure 4d to f). These results suggest that strain competitiveness for nodulation formation is relatively robust to the presence of additional strains, and strains with higher frequency in pairwise competitions will also have higher frequencies in more complex communities. Interestingly, we did observe hints of negatively frequency dependence; extremely low-frequency strains tended to be more frequent in more complex communities (e.g., M162 in C3 versus C68 and T073 in C8 versus C68; Fig. 4c and f).

**TABLE 2:** Rankings of strain frequencies in two strain communities were consistant across increasingly complex communities: three strains (C3), eight strains (C8), and 68 strains (C68)^*a*^

Host	Pairwise community	Pairwise strain frequency (4 reps)	C3 (5 reps)	C8 (5 reps)	C68 (6 reps)
A17	**HM006.1**:KH46c	**0.79**:0.21	1	1	1
	M162:**HM006.1**	0.01:**0.99**	1	1	1
	M162:**KH46c**	0.01:**0.99**	1	1	1
R108	**HM006.1**:KH46c	**0.56**:0.44	0.83	0.66	1
	M162:**HM006.1**	0.01:**0.99**	1	1	1
	M162:**KH46c**	0.01:**0.99**	1	1	1

a The higher frequency strain is shown in bold followed by the proportion of replicates in which the higher frequency strain also had higher relative frequency in the more complex communites.

### Host-specific effects of N, density, and complexity on nodule and plant traits.

The effect of N addition and inoculum density on plant phenotypes were host specific (Table S3). In A17, increased rhizobial density resulted in plants forming more nodules (*P = *0.004; Fig. 3a) that weighed less (*P < *0.001; Fig. 3b). In R108, an increase in rhizobial density resulted in plants forming more, smaller nodules, but only in the N-addition environment (Nitrogen × Density: *P = *0.036 and *P = *0.033, respectively). Neither N addition nor rhizobial density had measurable effects on either above or belowground biomass. However, both N (*P = *0.029) and to a lesser extent density (*P = *0.087) decreased the root to shoot ratio in A17 but not in R108 (Fig. S7). The complexity of the inoculum community had little effect on nodule number or vegetative biomass (Fig. S8 and Table S4), although it did have minor effects on nodule weight in A17 but not R108 (Host × Complexity: *P* = 0.018) and root weight (*P = *0.053) of both hosts.

## DISCUSSION

The symbiosis between legumes and rhizobia plays an important ecological role by contributing nitrogen to natural and agricultural ecosystems (65–67). Here we asked how host genotype and each of three environmental factors likely to vary spatially and temporally affect the rhizobial community in nodules. Despite being the key benefit that rhizobia provide plants, nitrogen (at least at the low addition level used here) had relatively minor effects on the strain composition, diversity, and predicted benefit of nodule communities. Similarly, the complexity of the inoculum community (number of strains used to inoculate a plant) had only minor effects on relative strain rankings, suggesting that higher-order interactions among strains do not alter the pairwise rankings of strain success in the context of nodule formation and development. In contrast, a 100-fold change in inoculum density had large and host-specific effects on the nodule community. In host A17, density had a strong effect on strain composition, diversity, and benefit and thus potentially selection on the rhizobial population. We caution however that our results are based on only two N and density levels and plant genotypes. Moving forward, it will be interesting to expand investigations to include the broad range of rhizobia population sizes, nitrogen levels, and legume genotypes and species found in natural and agricultural systems.

Theoretically, the availability of alternative sources of a symbiotic resource can alter selection on beneficial symbionts (30, 31, 68). When mutualistic relationships are considered in a market framework, external N improves the bargaining power of the host and somewhat counterintuitively selects for strains that “negotiate” more strongly and thus are less beneficial strains (31). In contrast, other models suggest that relaxed selection for symbiont benefit could occur because it is less costly for the host to allocate resources to a less beneficial partner if it can obtain ample resources from an alternative source (30). Despite the potential, we found that the addition of small amounts of N had little effect on rhizobial strain composition, diversity, and the host benefits of the nodule community. Indeed, in one host genotype (A17), selection for beneficial strains was stronger, not weaker, in the N-addition environment. Our findings of subtle effects of N addition are consistent with several other studies that have examined the composition of strains that form nodules (37–40, 69). Similarly, our results align with two studies that assessed composite strain fitness across all nodules formed by a plant. Nitrogen addition did not affect rhizobial composition in pools of 4-week-old *Medicago* nodules inoculated with a mixture of two *Sinorhizobium* species or a mixture of Fix− and Fix+ strains (39) and had weak effects on strain fitness of two species of *Acacia*; drought and phosphorous addition treatments had larger effects (24).

Although our results are consistent with these short-term (single growing season) studies, it is clear the addition of high levels of N over longer periods of time can affect the symbiosis. Many legumes regulate nodule formation and symbiotic nitrogen fixation when high levels of abiotic nitrogen are available, presumably reflecting the cost of forming and maintaining the symbiosis (33). For instance, Weese et al. (70) and Klinger et al. (71) found rhizobia in a grassland community to be less beneficial to hosts after 22 years of N addition. One way to reconcile discrepancies between short and long-term experiments is to note that other parameters that influence absolute fitness could change even if relative fitness is not altered by nitrogen addition. For instance, reductions in host population sizes, nodule numbers, or nodule size could all increase the importance of rhizobial adaptation to environments external to the host and result in a population of strains with reduced host benefit via selective tradeoffs or drift (18, 28, 72–74). To test such a hypothesis will require follow-up experiments (i) at a broader range of nitrogen-addition levels and (ii) that vary rhizobial population densities and frequency of exposure to host and nonhost environments.

Whereas the effects of nitrogen on the legume-rhizobia symbiosis have been the focus of many studies, the potential for density-dependent selection shaping legume-rhizobia symbiosis has received scant attention. Nevertheless, rhizobial density varies widely in nature (e.g., 10^2^ to 10^6^ per gram; Yan et al. [44]). The 100-fold difference in inoculum density we examined resulted in host-dependent shifts in the composition of the nodule community, with increased density resulting in more beneficial strains being favored in one host and less beneficial strains being favored in the other host. While the underlying mechanisms for this pattern remain to be identified (e.g., quorum sensing/chemotaxis), the results raise questions about the generality of laboratory experiments that rely on extremely high densities of rhizobia, presumably to ensure that hosts will not be rhizobia limited. Interestingly, while individual nodules sampled from field-grown plants are nearly always occupied by a single rhizobial strain (69, 75, 76), the probability of one nodule being infected by multiple strains increases with rhizobial density (49, 50); however, at extremely high densities, quorum-sensing mechanisms shut down the rhizobial response to hosts. Whether strain competition occurs via direct interactions within a nodule versus competition between nodules inhabited by different strains can affect mechanisms of rewards/sanction and the scale at which selection occurs (77) is yet to be determined.

Although most experiments are conducted with relatively simple rhizobial communities, in nature legume hosts are exposed to many strains (44, 52) as well as other microbes that might affect the establishment of the symbiosis (23, 78). Studies of species-level interactions between microbes show that adding an additional species can sometimes modify interactions between two species and even cause transitions from mutualisms to antagonisms (79). We did not find evidence for these sorts of shifts among S. meliloti strains. The rank order relationships among focal strains were mainly unchanged when we added additional strains. Because we know of no other studies of mutualism that examine intraspecific bacterial variation, it is not easy to contextualize these results. Still, the apparent lack of shifts in pairwise interactions indicates that mixed-strain experiments could help classify strain interactions more broadly. For instance, a similar methodology could be used to assess competitive outcomes between multiple mutant strains and a wild-type strain in a single experiment instead of conducting many pairwise competition experiments. Similarly, our results suggest that multistrain inoculation experiments could be used in breeding pipelines to screen for rhizobial strains with high fitness on specific host varieties.

Experimental evolution studies, such as this one, allow assessment of the relative importance of deterministic and stochastic processes in species interactions (80–82). The two host genotypes differed markedly in the characteristics of nodules in ways that could influence the evolution of rhizobial symbionts. For instance, A17 made many small nodules while R108 made a few large nodules, a pattern found in all nitrogen, density, and inoculum environments. Despite these differences, overall investment in nodule tissue per plant was very similar for both hosts. Decreases in nodule numbers at low inoculum densities were offset by increases in nodule size (a pattern observed by Singleton and Tavares [83]). While rarely discussed from the rhizobial perspective, these striking genotypic differences could have significant consequences for the evolution of rhizobial symbionts. Our observation of low nodule numbers and low selectivity of R108 host plants provides a testable hypothesis for the increased variability in strain fitness among replicates. Increased stochasticity in strain fitness outcomes even when pooling nodules from 10 or more host plants could influence rhizobial evolution, not by directly determining rhizobial fitness, but by increasing the strength of drift. Our results add to the increasing number of studies documenting the potential importance of stochastic processes (84–86) in host-microbe interaction and hint that genetically influenced host traits can drive differences in the relative importance of these processes. Screening additional accessions of Medicago truncatula for these traits and expanding the scale of genetic variation to individual host genes and multiple Medicago species will provide a broader understanding of the range of host genetic control on symbiont evolution.

### Conclusions.

Using a high-throughput methodology, we evaluated the context dependence of host-dependent strain fitness in legume-rhizobia symbiosis. Our finding that host genetic variation is a consistent driver of rhizobial relative fitness across environmental perturbations suggests it is possible to identify the genomic basis of strain × host interactions (87–89) and use that information to identify successful rhizobial strains even when environmental conditions change across years or locations. Our results also suggest that the selection hosts impose on rhizobial populations might depend on the density of the rhizobial population in the soil. From a translational perspective, understanding density-dependent selection could aid in developing beneficial inoculants that overcome establishment barriers (90).

## MATERIALS AND METHODS

### Constructing communities.

To form the rhizobial communities, we grew each of 68 strains in 3 mL Tryptone yeast media (6 g tryptone, 3 g yeast extract, 0.38 g CaCl_2_/L) for 3 days and then combined an equal volume of each culture to generate a community (C68) with approximately equal representation of each strain (median strain frequency: 0.014; range: 0.009 to 0.02). We used the same method to form the eight (C8), three (C3), and three two-strain communities (M:H, M:K, and H:K). To directly measure the strain frequency in each initial community, we extracted and sequenced four replicates of each community using the same method detailed for nodules (see Strain frequencies). All strain names are listed in Fig. 2d.

### Experimental details.

Seeds of two host genotypes A17 var. Varma and R108 (Medicago HapMap accession numbers HM101 and HM340; Stanton-Geddes et al. [96]) were bleached, rinsed, scarified with a razor blade, stratified on wet filter paper at 4°C in the dark for 2 days, and then allowed to germinate at room temperature for 1 day. We chose these genotypes because they (i) are commonly used for molecular genetics work and (ii) differ in nodule strain communities, transcriptomes, and symbiotic traits. Twelve germinated seeds were then planted in each of 45 1-L pots filled with sterilized Sunshine Mix. When seedlings were 3 days old, 100 μL of each rhizobial community diluted in 9.9 mL 0.85% NaCl wt/vol solution were used to inoculate each pot (approximately 10^8^ cells, except for the low-density treatment, which was inoculated with ~10^6^ cells; Fig. S1). We inoculated 6 replicate pots for each C68 treatment, 5 for C8, and 4 for each pairwise community (Fig. 1). Plants were fertilized with 150 mL of N-free fertilizer (Bucciarelli et al. [91] see Burghardt et al. [28] for details) once a week and watered with sterile water as needed. 6 weeks after planting, we sampled ~300 to 500 (A17) or ~100 to 200 (R108) nodules from the plants in each pot (Fig. S9). Because genotype A17 produces 5 to 10 times more nodules than genotype R108, this sample represents nodules from all 10 to 12 plants in pots with R108 and ~6 plants from A17 pots. This nodule pool size is designed to ensure we have statistical power to overcome stochastic processes underlying nodule formation, given the 68 potential strain partners. Nodule pools were crushed, and we used a series of differential centrifugation steps to enrich for and pellet undifferentiated bacteria (Burghardt et al. [28]). In *Medicago* species, undifferentiated rhizobia are the fraction of interest for measuring rhizobial fitness because these rhizobia, not terminally differentiated bacteroids, are released back into the soil when nodules senesce. Pellets were stored at −20°C until we extracted DNA using the UltraClean Microbial DNA isolation kit (no. 12224; Mo Bio Laboratories). In addition to harvesting the nodules, we measured on a per plant basis: nodule number, nodule fresh weight, and vegetative and root biomass (dried at 60°C for 72 h). For each host genotype, we sampled six nodule pools for the four Nitrogen × Density C68 treatments, five nodule pools for the C8 and C3 treatments, and four nodule pools for each of the three pairwise community treatments.

### Strain frequencies.

We estimated the frequency of each strain in each nodule pool using the method in Burghardt et al. (28). In brief, DNA isolated from each replicate was sequenced on an Illumina HiSeq 2500 (NexteraXT libraries; 125-bp paired-end reads and 3.6 to 9.4 million read pairs library^−1^). Reads were trimmed with TrimGalore! (v0.4.1) using default settings, except with minimum read length: 100 bp; quality threshold: 30; and minimum adapter match: 3. We used bwa mem (v0.7.17; Li and Durbin [92]) with default settings to align reads to the *Sinorhizobium (Ensifer) meliloti* USDA1106 genome (Nelson et al. [59]; NCBI BioProject: PRJNA388336). After cleaning and alignment, the median read depth per sample was 65× (range: 29× to 115×). Using prior genome sequencing data, we identified single nucleotide polymorphisms (SNPs) segregating in each of the sequenced communities using FreeBayes (v1.2.0-2-g29c4002; reference 93) with a minimum read mapping quality of 30. All 68 strains used here differ at more than 1,000 SNPs which, based on our simulations, provides ample power for strain frequency reconstruction. To estimate strain frequency, we used only SNPs for which every strain had an unambiguous call. We then estimated the frequency of each strain in each sample using HARP (94) as described in Burghardt et al. [28]. Briefly, this method estimates the likelihood that each read comes from each strain and summarizes this signal across all reads to estimate strain frequencies.

### Nodule community measurements.

Based on strain frequencies, we calculated three nodule community metrics for each replicate pot: composition, diversity, and host benefit. We estimated strain composition as the fold change in the frequency of each strain (*q_x_*) in a nodule community relative to the mean frequency of that strain across four sequencing replicates of the initial community [fitness = log_2_(*q_x_*_selected/_*q_x_*_initial_)]. This transformation normalizes the frequency distribution and controls for small differences in initial strain frequency (median *q_x_*_initial_ = 0.0092, 5% to 95% quantile: 0.005 to 0.0154). We estimated community diversity as the exponent of Shannon diversity, which we calculated using the “renyi” function in the vegan package of R. We calculated predicted host benefit as the sum of the per strain frequency in nodules multiplied by the dry plant weight from a single-strain inoculation experiment involving both A17 and R108 (data from Burghardt et al. [28]):

$$\text{Host benefit}=\frac{\sum_{x=1}^{n}q_{x}*\mathrm{dry}\;\mathrm{plant}\;\mathrm{weight}\;\mathrm{in}\;\mathrm{single}\;\mathrm{strain}\;{\mathrm{experiment}}_{x}}{\sum_{x=1}^{n}q_{x}}$$

We scaled each host-specific data set from zero (full occupancy by the least beneficial strain) to one (full occupancy by the most beneficial strain). We omitted strains for which we did not have single-strain plant weight estimates (9 strains for A17 and 29 strains for R108). While missing strains may reduce the power to make between host comparisons, missing strains span the phylogeny, and there is no reason to suspect that our subsamples are biased.

### Statistical analysis of community measures.

We used redundancy analysis (RDA; “rda” in the vegan R package; Oskanen et al. [95]) and ANOVA to analyze the effects of Host genotype (H), Density (D), and Nitrogen (N) and their interactions on each of the nodule community measures. To collapse the dimensionality of the strain relative fitness data and analyze shifts in relative fitness across treatments, we used RDA. RDA fits a multivariate linear regression to centered and scaled data and then uses PCA to decompose the major axes of variation in the fitted parameters. The adjusted R^2^ of each RDA model provides an estimate of the proportion of variance in relative fitness explained by the model predictor(s). We permuted the data to determine the probability that fitness differences occurred by chance (“anova” function, 999 permutations). We used an ANOVA (“lm” and “anova”) to analyze strain diversity to test for differences among treatments. To analyze host benefit, we used an ANOVA (“lm” and “anova”). Because the effects of D and N were host dependent (H × D, H × N interactions), we also analyzed the effects of N, D, and their interaction for each host separately.

### Analysis of rank order and community complexity.

For each pairwise competition of the three strains used to form a three-strain community (C3), we identified the strain with higher strain frequency and asked if the same strain was at higher frequency in the more complex three strain community. We also examined whether strain frequency rankings in the C3 community remained the same in the 8 (C8)- and 68 (C68)-strain communities. Likewise, we examined whether the frequency rankings of each of the strains in the C8 community remained the same in the C68 community.

### Statistical analysis of plant phenotypes.

We used an ANOVA (“lm” and “anova”) to test for the effects of host genotype, inoculum density, and nitrogen level and their interactions on six plant traits (nodule number, average nodule weight, nodule weight per plant, shoot biomass, root biomass, and root to shoot ratio). We collected these data for each plant growing in each pot, and then we used the mean value per pot for subsequent analyses. To control for slight differences (10–12) in plant number per replicate pot, these traits are analyzed as per plant means. The first two traits violated the assumption of homogeneity of variance between hosts, so we focused on host-specific analyses. To evaluate the effect of community complexity on plant phenotypes, we ran a model with Host (H) and community complexity (C; 2, 3, 8, and 68 strains) and their interaction as continuous predictors. Although we test for the among-treatment differences in plant phenotypes, the experiments were designed to evaluate the effect of treatments on strain fitness. They were not well powered to detect among-treatment differences in plant phenotypes.

### Data availability.

The data supporting the results of this study and the code necessary for these analyses are available on a GitHub repository (https://github.com/BurghardtLab/NitrogenxDensityxComplexity-SelectandReseq). The raw reads have been deposited in NCBI BioProject PRJNA401437. The NCBI accession numbers for the samples are SRR7699552 to SRR7699563, SRR7699565 to SRR7699576, SRR7699578 to SRR7699607, SRR7699611, SRR7699614, SRR7699616 to SRR7699619, SRR7699622, SRR7699623, SRR7699625 to SRR7699631, SRR7699635 to SRR7699654, SRR7699657 to SRR7699659, SRR7699661 to SRR7699664, SRR7699666 to SRR7699668, SRR7699670, SRR7699672 to SRR7699685, and SRR7699687 to SRR7699694.
